# Clinical Review: Navitoclax as a Pro-Apoptotic and Anti-Fibrotic Agent

**DOI:** 10.3389/fphar.2020.564108

**Published:** 2020-11-26

**Authors:** Nur Najmi Mohamad Anuar, Nur Syahidah Nor Hisam, Sze Ling Liew, Azizah Ugusman

**Affiliations:** ^1^ Programme of Biomedical Science, Centre for Toxicology & Health Risk Studies, Faculty of Health Sciences, Universiti Kebangsaan Malaysia, Kuala Lumpur, Malaysia; ^2^ Department of Physiology, Faculty of Medicine, Universiti Kebangsaan Malaysia Medical Centre, Cheras, Malaysia

**Keywords:** anti-cancer agent, apoptosis, cancer, fibrosis, navitoclax (ABT-263), B-cell lymphoma

## Abstract

B-cell lymphoma 2 (BCL-2) family proteins primarily work as a programmed cell death regulator, whereby multiple interactions between them determine cell survival. This explains the two major classes of BCL-2 proteins which are anti-apoptotic and pro-apoptotic proteins. The anti-apoptotic proteins are attractive targets for BCL-2 family inhibitors, which result in the augmentation of the intrinsic apoptotic pathway. BCL-2 family inhibitors have been studied extensively for novel targeted therapies in various cancer types, fibrotic diseases, aging-related as well as autoimmune diseases. Navitoclax is one of them and it has been discovered to have a high affinity toward BCL-2 anti-apoptotic proteins, including BCL-2, BCL-W and B-cell lymphoma-extra-large. Navitoclax has been demonstrated as a single agent or in combination with other drugs to successfully ameliorate tumor progression and fibrosis development. To date, navitoclax has entered phase I and phase II clinical studies. Navitoclax alone potently treats small cell lung cancer and acute lymphocytic leukemia, whilst in combination therapy for solid tumors, it enhances the therapeutic effect of other chemotherapeutic agents. A low platelet count has always associated with single navitoclax treatments, though this effect is tolerable. Moreover, the efficacy of navitoclax is determined by the expression of several BCL-2 family members. Here, we elucidate the complex mechanisms of navitoclax as a pro-apoptotic agent, and review the early and current clinical studies of navitoclax alone as well as with other drugs. Additionally, some suggestions on the development of navitoclax clinical studies are presented in the future prospects section.

## Introduction

Navitoclax is one of the B-cell lymphoma 2 (BCL-2) family protein inhibitors which has been generated during the clinical development of ABT-737 (Tse et al., 2008). This orally bioavailable drug, which was formerly known as ABT-263, has a similar structure to its predecessor, ABT-737. Three main sites of ABT-737 have been identified to cause its large molecule (molecular weight >800 g/mol) and contribute to its poor profile in terms of charge balance, affinity as well as metabolism (Park et al., 2006; Tse et al., 2008). The results from ABT-737 preclinical studies have proven its poor bioavailability and physicochemical properties which then caused the clinical studies to be impeded (Tse et al., 2008). Modification of ABT-737 structure to ABT-263 aims to maximize the drug potency, pharmacokinetics and pharmacodynamics (Bruncko et al., 2007), hence increasing its potential as an anti-cancer drug. Additionally, early studies on navitoclax have shown promising results to suppress tumors in small cell lung cancers (SCLC) and acute lymphocytic leukemia (ALL) (Tse et al., 2008). One study has demonstrated a high affinity profile of navitoclax toward BCL-2 family proteins (Chen et al., 2011). Navitoclax is a known BH3 mimetic drug and potently binds to the BH3 domain of BCL-2 anti-apoptotic members. Upon administration, navitoclax binds to the BH3 binding groove of BCL-2 proteins which are located in the cytoplasm, causing the displacement of pro-apoptotic BH3-only protein, BIM, from BCL-2 (Mérino et al., 2012). BIM is then set free to trigger the release of small heme proteins, the cytochrome c, from mitochondria causing cell apoptosis (Mérino et al., 2012).

**FIGURE 1:**
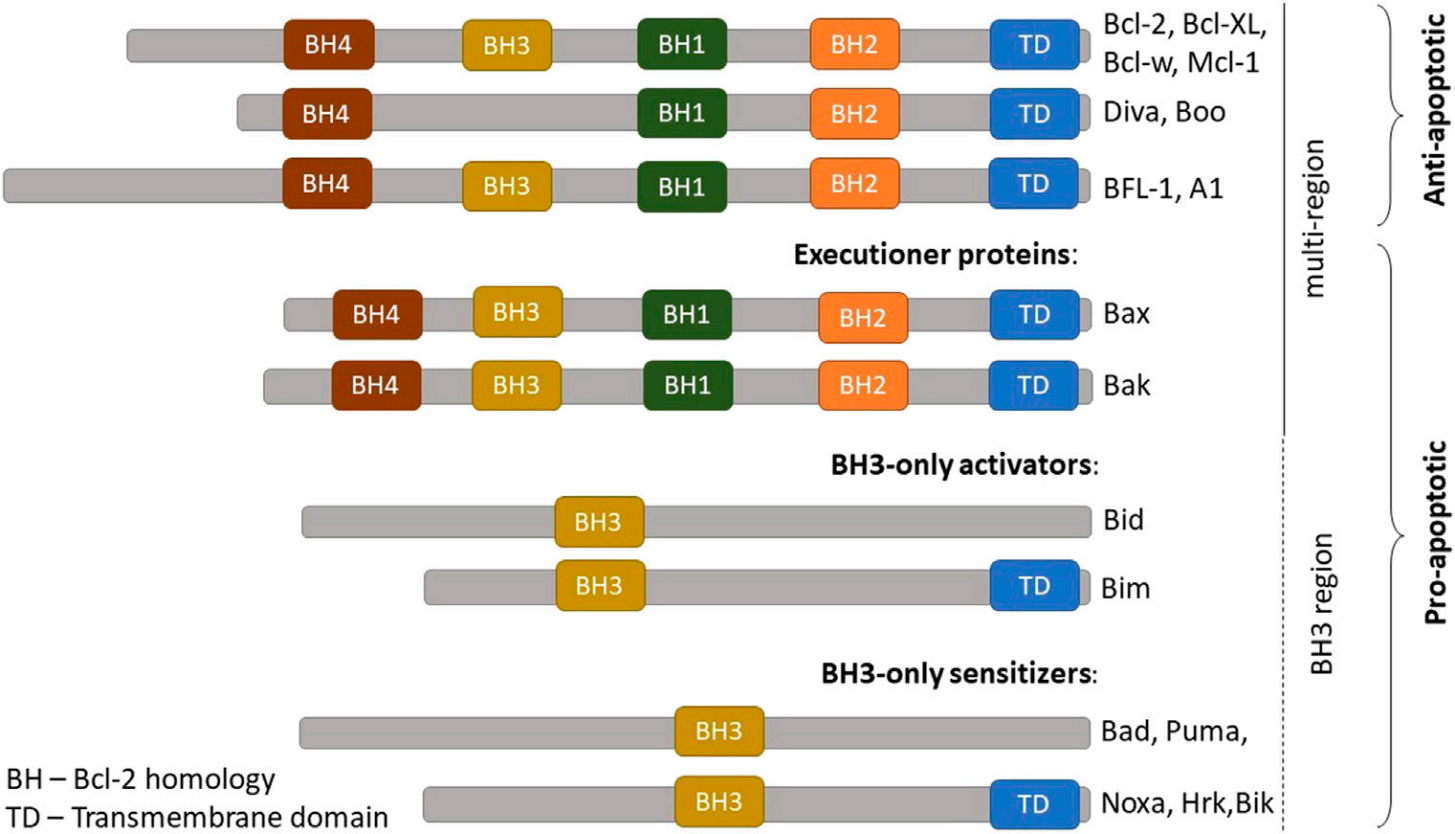
BCL-2 family protein members. The BCL-2 family members are comprised of anti-apoptotic and pro-apoptotic proteins. Pro-apoptotic proteins are further categorized into three groups according to their functions: **(**A) Pro-apoptotic effectors = executioner proteins **(B)** Proapoptotic activators = BH3-only activators **(C)** Proapoptotic sensitizers = BH3-only sensitizers. BCL-2, B-cell lymphoma 2; BCL-X_L_, B-cell lymphoma-extra-large; MCL-1, Myeloid cell leukemia; A1, BCL-2 related protein A1. Adapted from Shamas-Din et al. (2013).

**FIGURE 2:**
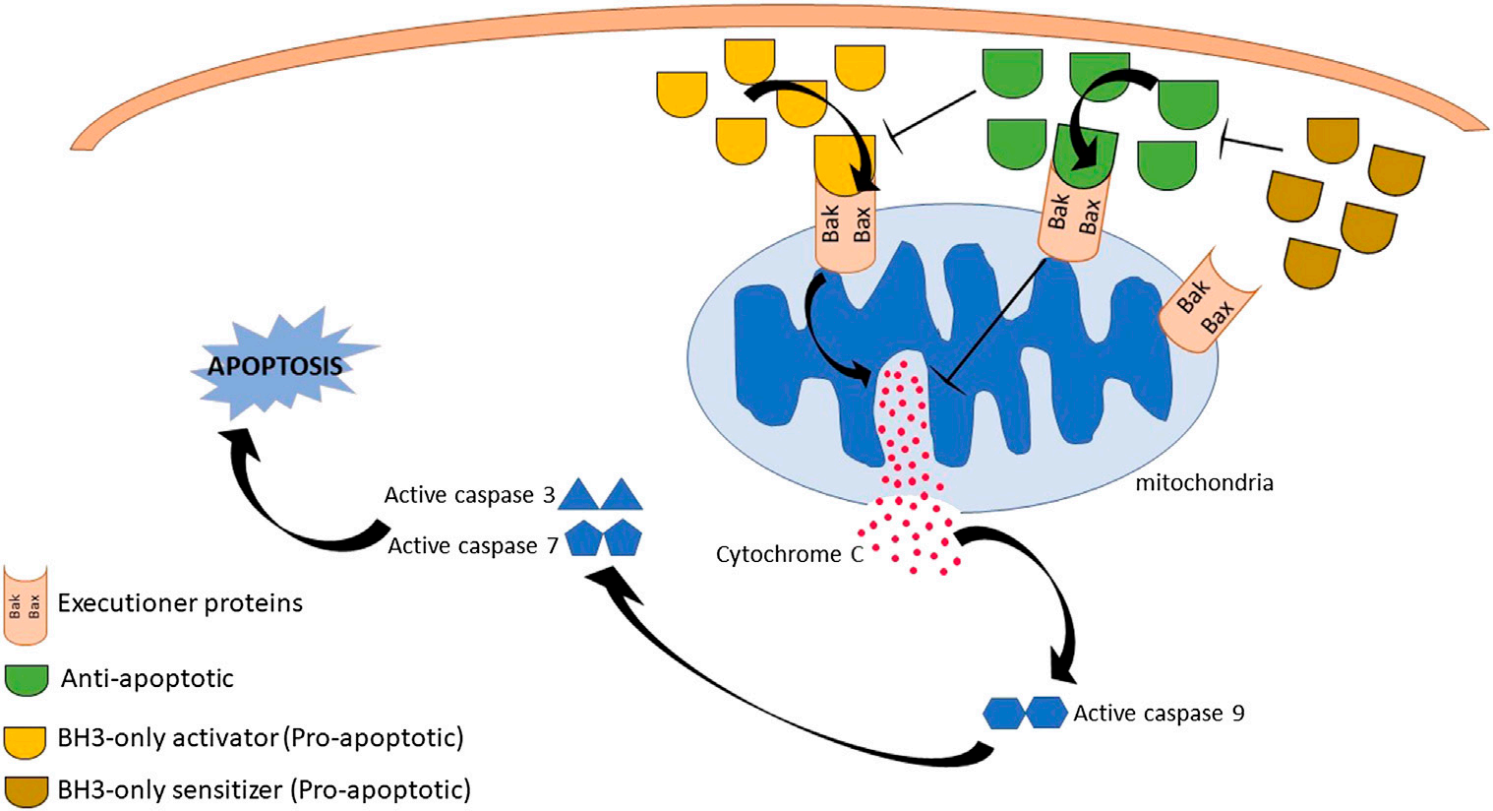
Summary of B-cell lymphoma 2 (BCL-2) family proteins mechanism of actions. Initiation of death signal is through the binding of BH3-only proapoptotic proteins either into the hydrophobic groove of antiapoptotic BCL-2 family or executioner proteins, leading to executioner proteins activation and oligomerization. This results in mitochondrial outer membrane permeabilization, cytochrome c release and activation of caspase activity. Eventually, cell death is observed. However, cell apoptosis is inhibited by anti-apoptotic proteins through the suppression of executioner proteins activities and blocking cytochrome c released.

The BCL-2 family members are categorized into three groups which represent their respective structure and function (Figure 1): i) Executioner proteins: stimulate apoptotic events directly or indirectly through BH3 proteins signaling that are able to detect cellular stress; ii) BH3-only proapoptotic proteins: regulate apoptosis by recruiting the executioner proteins, BAX or BAK, to oligomerize and trigger mitochondrial outer membrane permeabilization (MOMP); iii) Anti-apoptotic proteins: inhibit apoptosis by hindering BH3 and executioners proteins activities (Visvader et al., 2009; Shamas-Din et al., 2013). The activity of BCL-2 family members is a central regulator for cell survival or cell death. A brief description of their mechanism in modulating the intrinsic apoptotic pathway is illustrated in Figure 2.

Generally, the BCL-2 family protein structure consists of several BCL-2 homology (BH) domains which play a crucial role in activating cell apoptosis (Ibrahim et al., 2016; Tan et al., 2016). In fact, previous studies have shown that apoptotic event rates, as well as their interactions with other proteins signaling were affected by these domains alteration or deletion (Reed et al., 1996; Kawatani and Imoto, 2003; Monaco et al., 2013). Besides, the roles and interactions of BCL-2 family members to modulate programmed cell death are diverse and complex (Petros et al., 2004; Chen et al., 2005; Willis et al., 2005; Moldoveanu et al., 2014). An early study has discovered that some BH3-only proteins (i.e., BIM and PUMA) displayed random binding to all the anti-apoptotic proteins with comparable potency whilst others (e.g., BAD and NOXA) were observed to be selective (Chen et al., 2005). For example, BAD displayed high potency toward BCL-2, B-cell lymphoma-extra-large (BCL-X_L_) and BCL-W but low potency to A1 and no association with myeloid cell leukemia (MCL-1). In contrast, NOXA only showed interaction with MCL-1 and A1 (Chen et al., 2005). Concurrent selective binding of Bad and NOXA to their respective antiapoptotic BCL-2 proteins exhibited a potent stimulation of cell death (Chen et al., 2005). This selectivity is influenced by the differences in the pro-apoptotic activity of BH3-only members and certain selective interaction combinations that lead to complementary apoptotic function (Chen et al., 2005). Another study has reported that different anti-apoptotic proteins regulate the activity of executioner proteins in causing cell death. The study demonstrated that BAK activation was negatively regulated by the binding of MCL-1 and BCL-X_L_ only, whilst BAX activity was influenced by most of the anti-apoptotic members (Willis et al., 2005). Nevertheless, BAK inactivation could be reversed by the concurrent interaction of BH3-only proteins with MCL-1 and BCL-X_L_, thus restoring BAK-mediated apoptosis activity (Willis et al., 2005). This evidence has clearly illustrated the multicomplex molecular interaction of BCL-2 family members in modulating programmed cell death. In spite of that, the determination of several BCL-2 family proteins’ selectivity and specificity interactions could potentially contribute to the development of BCL-2 inhibitors.

Over the decade, further investigations and clinical studies involving navitoclax in cancer treatment have been carried out to evaluate its efficacy and toxicology. In addition, many existing investigations attempt to explore the apoptotic effect of navitoclax on other diseases such as chronic lymphocytic leukemia, epithelial cancer, breast cancer and fibrosis. Nevertheless, a comprehensive review of navitoclax pharmacological properties has yet to be presented. This review aims to clearly elucidate and compile the potential therapeutic use of navitoclax on various cancer types, tumor progression, and fibrosis. The discussion will center on the mechanisms and available clinical reports of navitoclax in treating those diseases in order to evaluate its pharmacological profile as well as tolerability in patients. Furthermore, the combination therapy of navitoclax with other drugs will also be reviewed. Lastly, suggestions on how to improve navitoclax clinical studies and other potential targets for navitoclax treatment will be included in the future prospects section.

## Role of Navitoclax on Cancer and Tumor Progression

**FIGURE 3:**
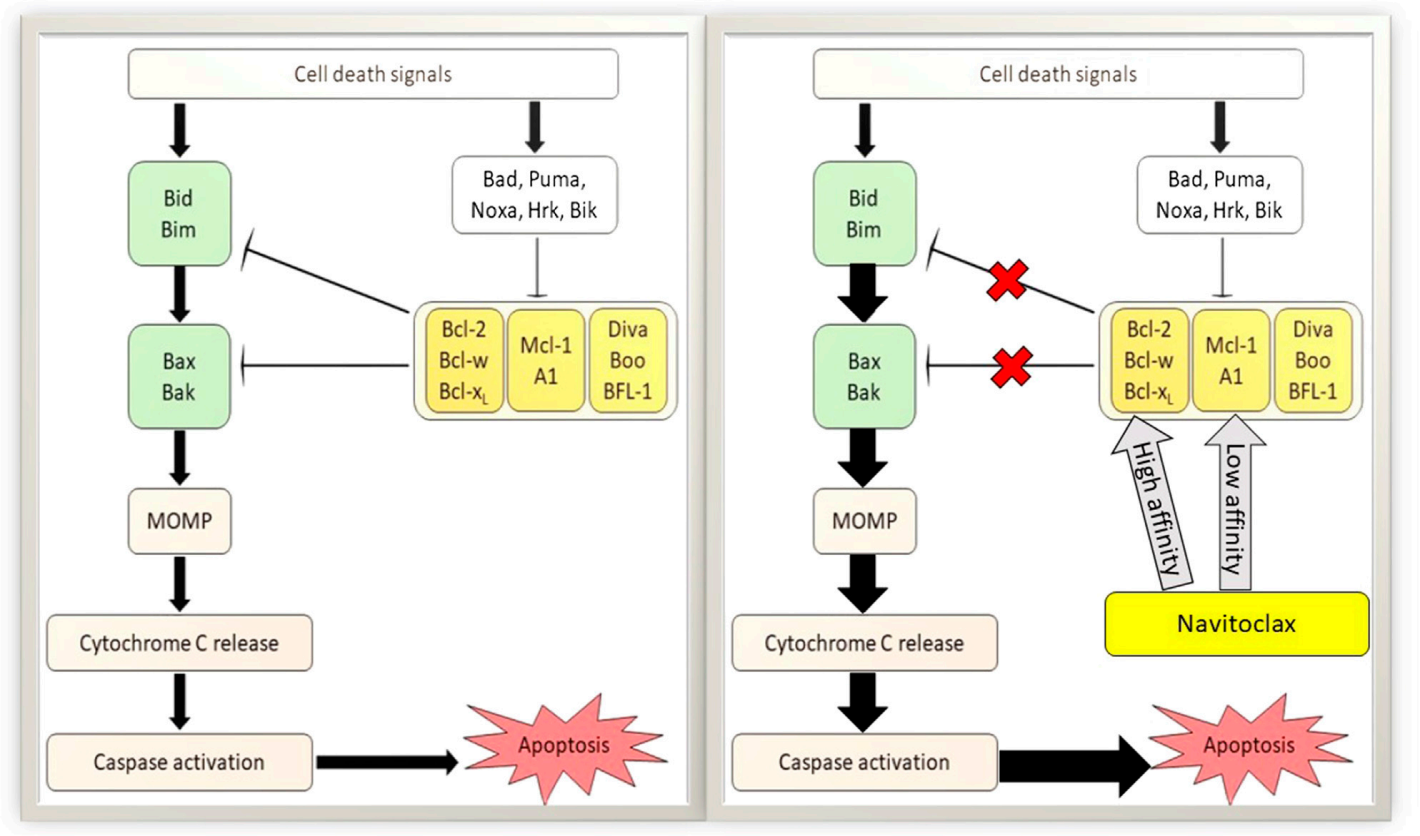
Apoptotic mechanism of navitoclax on tumor cells. Left: Classical apoptotic pathway of B-cell lymphoma 2 (BCL-2) family proteins. Right: Potentiation of apoptotic activity in the presence of navitoclax. Navitoclax affinity on antiapoptotic BCL-2 proteins are varied. MOMP, mitochondrial outer membrane permeabilization.

Uncontrolled cell growth and its spreading are the prime factors of cancer pathophysiology. Modulation of the intrinsic apoptotic pathway by BCL-2 family proteins highly influences the survival of cancer cells. Previous studies have demonstrated an abundant expression of anti-apoptotic proteins, commonly BCL-2, BCL-X_L_, and BCL-W in multiple myeloma cell lines (Gazitt et al., 1998) and non-SCLC tissues (Liu et al., 2017) which are associated with tumor advancement, sustenance (Liu et al., 2017) and chemoresistance (Gazitt et al., 1998; Shangary et al., 2004; Paulus et al., 2014). Avoidance of apoptosis through the promotion of pro-survival BCL-2 family proteins is effective to support the pathogenesis of cancer. Meanwhile, a stimulation of intrinsic apoptosis after DNA damage would enhance the potency of chemotherapeutic agents (Gandhi et al., 2011). One of the strategies for cancer treatment development is via promoting the intrinsic programmed cell death. Blocking the interaction of anti-apoptotic proteins with executioner proteins on mitochondrial membranes has been the basis for that approach. Therefore, the pro-survival proteins of BCL-2 families are the potential targets for this therapeutic intervention. Several BCL-2 family inhibitors have been explored as anti-cancer drugs and one of them is navitoclax. A study has reported the mechanism of navitoclax to induce cancer cell apoptosis by disrupting the interaction of anti-apoptotic proteins with BH3 domain binding proteins as shown in Figure 3. As a consequence, the free BH3-only activators initiate BAX translocation resulting in mitochondrial MOMP (Han et al., 2019). MOMP leads to the cytochrome c secretion from the mitochondrial intermembrane space into the cytoplasm (Bender and Martinou, 2013) and further stimulate downstream signaling of intrinsic apoptosis through caspase proteins’ interaction. Ergo, cancer cells further proliferation are abolished, and in some cases, this may promote the chemotherapeutic regimens. Nevertheless, the mechanism of navitoclax in mediating anti-tumor activity of various cancer types by recruiting BCL-2 family proteins is complex and yet to be well elucidated. The following are the studies of navitoclax therapy on various cancer types, mostly aimed to explore its mechanism, efficacy, side effects, pharmacodynamics as well as pharmacokinetic profiles.

### Navitoclax Clinical Studies on Various Cancer Types

#### Preclinical Studies

*In vitro* and *in vivo* studies conducted by Tse et al. (2008) were the first to show the inhibitory effect of navitoclax on anti-apoptotic proteins of the BCL-2 family for various tumor therapies including SCLC. Navitoclax was found to have a high affinity toward BCL-2 and BCL-XL proteins, but not to MCL-1 protein (Tse et al., 2008). The *in vitro* study has further confirmed the navitoclax interaction with BCL-2 protein, as this drug weakly induced apoptosis of BCL-2-deficient cells (Tse et al., 2008). Furthermore, their study demonstrated the downstream signaling of navitoclax through BAX activation and the release of cytochrome c (Tse et al., 2008). *In vivo* study on SCLC xenograft models found that daily oral dosing of navitoclax effectively attenuates tumor progression (Tse et al., 2008). Dosages of 25–50 mg/kg have induced tumor suppression in almost half of the models studied and even with a low dosage, a moderate tumor inhibition was observed. Their steady-state pharmacokinetics study demonstrated that approximately 5.4–7.7 μmol/L of navitoclax peak plasma concentrations are of high potency (Tse et al., 2008). Other than that, a further study conducted by Shoemaker et al. (2008) aimed to elucidate the efficacy of navitoclax in several SCLC cell lines (*in vitro*) and xenograft models (*in vivo*). Navitoclax efficacy on different SCLC xenograft models was varied, yet the treatment showed the induction of BAX translocation, cytochrome c release and caspase-3 stimulation were dose-dependent through the cellular level. Nonetheless, the results displayed a good correlation between *in vitro* cellular potency and *in vivo* tumor efficacy where the cell lines with the higher potency (EC50 <200 nmol/L) exhibited 100% objective response rate [ORR: the proportion of patients with tumor size reduction of a predefined amount and for a minimum time period (Kogan and Haren, 2008)] *in vivo* (Shoemaker et al., 2008). Dose regimen tests have demonstrated the high efficacy of navitoclax through continuous dosing (whether once or twice daily) compared to intermittent dosing (every 3 or 7 days). These findings indicate that acute administration of navitoclax is sufficient to significantly kill cancer cells. However, chronic treatment contributes to a stronger effect on tumor regression as well as delaying tumor growth (Shoemaker et al., 2008)

Navitoclax treatment also has been investigated for several lymphoid malignancies such as ALL, B-cell lymphoma and multiple myeloma. Even though these cancer types are quite similar in some ways as they are originated in the bone marrow, navitoclax exhibits different efficacy toward them. An *in vivo* study on an ALL xenograft model had demonstrated a potent anti-cancer effect of navitoclax (Tse et al., 2008). This result was supported by a pediatric preclinical testing program study, where a total of 23 panels of cell lines were treated with navitoclax at concentrations ranging from 1.0 nM to 10.0 µM, and the outcomes showed the greatest treatment sensitivity toward ALL cell lines (Lock et al., 2008). Similarly, *in vivo* results against ALL xenograft models also have displayed a significant therapeutic effect, but it was less sensitive against pediatric solid tumors despite the dosing and administration timing were according to the SCLC research procedure (Lock et al., 2008). Moreover, it is fascinating to observe that navitoclax has significantly potentiated the efficacy of some chemotherapeutic regimens (i.e., rituximab, doxorubicin, cyclophosphamide, vincristine, bortezomib and prednisone) in the xenograft models of other lymphoid malignancies including B-cell lymphoma and multiple myeloma (Tse et al., 2008). Nonetheless, single navitoclax treatments fairly inhibit the growth of these tumor types (Tse et al., 2008). Following this finding, Ackler et al. (2010) have conducted an investigation to assess potential augmentation of several common chemotherapeutic agents in blood cancer models by navitoclax. Both of these *in vitro* and *in vivo* studies demonstrated the ability of navitoclax to enhance other chemotherapeutic agents’ effects, significantly improve the ORR, massively attenuate tumor development and effectively delay the tumor growth (Tse et al., 2008; Ackler et al., 2010). Ackler et al. (2010) proposed that the upregulation of BCL-2 and BCL-XL in targeted tumor lines has developed resistance toward the common cytotoxicity agents in which the sensitivity could be restored in the presence of navitoclax.

A preclinical study by Shi et al. (2011) on a panel of epithelial cancer lines also demonstrated that MCL-1 and BCL-XL play a crucial role as apoptotic mediators during a prolonged mitotic arrest. BCL-2 family members' expressions are varied throughout the mitosis cycle. During interphase, the MCL-1 level is elevated, hence explaining the low responsiveness of epithelial cancer lines toward navitoclax treatment (Shi et al., 2011). While during mitotic arrest (developed by antimitotic drug), MCL-1 expression is low due to an imbalance between synthesis and proteolysis, thus allowing navitoclax to effectively accelerate apoptotic activity through inhibition of the BCL-XL signaling pathway (Shi et al., 2011). They also found that BIM upregulation is unnecessary to promote apoptosis during mitotic arrest, though other pro-apoptotic proteins expression should be determined in the future (Shi et al., 2011). Therefore, Shi et al. (2011) results have shed new light on epithelial cell-variation, whereby in cells with low BCL-XL levels (i.e., HeLa), downregulation of MCL-1 alone was sufficient to trigger BAX/BAK translocation and cause cell apoptosis. Whereas, in cells with substantial levels of BCL-XL (i.e., U2OS, OVCAR-5, A549), loss of MCL-1 alone was insufficient, but a combination with BCL-XL inhibitors such as navitoclax would cause BAX/BAK translocation that led to cell apoptosis (Shi et al., 2011). These data are the first to illustrate the point at which cell death is potentiated by navitoclax in the cell cycle phase and is governed by BCL-2 family members’ expressions.

Recent studies have reported the potency of navitoclax on breast cancer cells. However, the clinical trials were limited due to the occurrence of resistance toward monotherapy in breast cancer cells. Additionally, monotherapy with BCL-2 family did not induce tumor death in some breast cancer cell lines (Chen et al., 2011; Oakes et al., 2012; Vaillant et al., 2013). Jha et al. (2012) have reported that survivin is overexpressed in the majority of breast cancer cases, and its expression is associated with chemotherapeutic resistance. Thus, survivin-targeted therapy may be a good treatment approach for breast cancer patients (Lee et al., 2018). Primary functions of survivin include inhibiting cell apoptosis and regulating mitosis which is associated with the initiation of cancer formation (Lv et al., 2010). Lee et al. (2018) have conducted a study on two different types of breast cancer cell lines which are MDA-MB-231 and MCF-7. They reported different potencies of single navitoclax treatments on these cells. Navitoclax treatment alone had downregulated survivin expression and induced cell death in MDA-MB-231 cells, in contrast to MCF-7 cells that did not exhibit survivin reduction and causing cell resistance toward navitoclax (Lee et al., 2018). However, a combination therapy of navitoclax with a survivin inhibitor which is everolimus, was effective in stimulating an intrinsic apoptotic pathway in MCF-7 cells (Lee et al., 2018). Further *in vivo* studies of this novel polytherapy should be carried out in order to pursue clinical trials. Other than that, further preclinical studies using navitoclax with survivin inhibitors against various breast cancer cell lines could broaden the clinical data of navitoclax on breast malignancies (Lee et al., 2018).

Apart from that, Yang et al. (2019) have revealed the apoptotic activity of navitoclax as well as potential new molecular targets in human oral cancer-derived cell lines and mouse xenograft models. Their *in vitro* study has shown that navitoclax reduced the viability and stimulated cell death of HSC-3 and HSC-4 oral cancer cell lines (Yang et al., 2019). C/EBP homologous protein (CHOP) expressions were discovered to elevate in response to navitoclax treatment in a concentration- and time-dependent manner, thus indicating CHOP association with apoptotic activity of navitoclax in human oral cancer cells (Yang et al., 2019). CHOP is known as a critical element of the endoplasmic reticulum (ER) stress response and its expression is considered as an ER stress marker protein (Yang et al., 2017; Yang et al., 2019). Earlier studies demonstrated that several BCL-2 inhibitors may stimulate ER stress conditions, including accumulation and aggregation of unfolded and misfolded proteins to manifest their anti-tumor activities (Soderquist R. et al., 2014; Soderquist R. S. et al., 2014). Therefore, these studies support the findings of Yang et al. (2019) in suggesting that navitoclax works as an ER stress activator in mediating the apoptotic mechanism of oral cancer cells. In addition, an *in vivo* study using 100 mg/kg/day navitoclax treatment against oral cancer xenograft mice for 21 days found it exhibited a significant anti-tumor effect without apparent hepatic and renal toxicities (Yang et al., 2019). From these data, they postulated that navitoclax may be an attractive therapeutic drug candidate for human oral cancer therapy with CHOP as its alternative target in regulating the apoptotic activity and the side effects are also minimal. Hence, this study provides new insight into the navitoclax mechanism in regulating cell apoptosis through other families of apoptotic protein not only through BCL-2 family members. The findings from previous preclinical studies involving navitoclax in various cancer models are summarized in Table 1.

**TABLE 1:** Summary of navitoclax preclinical studies on various cancer types.

disease	Model	Dosage		Sample number	Side effect	Mechanism of action	References
Small cell lung cancer	*In vitro*: Human tumor cell lines	*In vitro*: 0 – 1,000 nmol/L		*In vitro*: SCLC cell lines → n = 22	Rapid but reversible thrombocytopenia	Navitoclax functionally inhibits BCL-X_L_ activity which then triggers BAX translocation and release of cytochrome, eventually causing cell apoptosis	Tse et al. (2008)
Lymphoid malignancies							
				Lymphoid malignancies cell lines → n = 23			
	*In vivo*: C.B. -17 *scid*-bg and C.B.-17 *scid* mice	*In vivo*: Mice → 100 mg/kg		*In vivo*: Mice → n = 8–10	—	—	—
	Beagle dogs	Dogs → 2 mg/kg/d (day 1 – day 6), then increased to 6 mg/kg/d (day 7 – day 12)		Dogs → n = 3	—	—	—
Hematologic tumors	B-cell lymphoma and multiple myeloma cell lines and mice models	*In vitro*: 0.1–100 nM		ND	Drug resistance	Navitoclax neutralizes BCL-2 activity, causing an increase of BAX oligomerization and subsequently enhances cell killing	Ackler et al. (2010)
		*In vivo*: 100 mg/kg/day					
Small cell lung cancer	*In vitro*: SCLC cell lines	*In vitro*: Various EC_50_ values of navitoclax have been determined for each SCLC cell lines tested, i.e., 110 nmol/L and 22 μmol/L against the most and the least sensitive cell lines respectively		*In vitro*: 30 cell lines	Transient thrombocytopenia and lymphopenia	Navitoclax inhibits BIM and antiapoptotic proteins interactions, which then stimulates the intrinsic apoptotic pathway. Moreover, navitoclax triggers cell apoptosis in cytokine-deprived cells through BCL-X_L_- and BCL-2-dependent signaling.	Shoemaker et al. (2008)
	*In vivo*: SCLC mice models	*In vivo*: 100 mg/kg/day and 50 mg/kg/day		*In vivo*: 11 mice models			
Solid tumors and hematologic malignancies	*In vitro:* Solid tumors and hematologic tumors cell lines		*In vitro:* 0.001–10.0 µM	*In vitro:* 23 cell lines	Thrombocytopenia	Navitoclax is positively correlated with BCL-2, BCL-W, BCL-X_L_ and with BH3-only proteins; BIM, whilst exhibiting negative correlation with MCL-1 expression	Lock et al. (2008)
	*In vivo*: Mice models		In – vivo: 100 mg/kg/day	*In vivo*: 44 xenograft mice models			
Epithelial cancer	Epithelial cancer cell lines		1 µM	ND	None	Navitoclax promotes cell killing during mitotic arrest is BCL-X_L_-dependent and is contributed by low MCL-1 expression. Plus, BH3-only proteins’ actions for cell apoptosis during mitotic arrest are dispensable	Shi et al. (2011)
Breast cancer	MDA-MB-231 and MCF-7 breast cancer cell lines		1 μM, 2 μM, 5 µM	ND	None	The single navitoclax treatment has induced apoptosis of MDA-MB-231 cell line, but not MCF-7 cell line. Stimulation of the apoptotic signaling are through survivin inhibition and caspase-activation-dependent	Lee et al. (2018)
Oral cancer	*In vitro*: Human oral cancer-derived cell lines; HSC-3, HSC-4, Ca9.22, HN22, MC-3, YD-15.		*In vitro*: 0 μM, 2 μM, 4 μM, 6 µM	—	None	Navitoclax acts as an endoplasmic reticulum stress inducer by upregulating C/EBP homologous protein (CHOP) expression to mediate apoptosis in human oral cancer cells	Yang et al. (2019)
	*In vivo*: Four-week-old female nude mice tumor xenograft models		*In vivo*: 100 mg/kg/day				

ND: not determined; BCL-X_L_, B-cell lymphoma-extra-large; SCLC, Small cell lung cancer.

#### Phase I Clinical Trials

A phase I clinical study has been carried out for a year on forty-seven patients with SCLC or pulmonary carcinoids by Gandhi et al. (2011). Intermittent and continuous navitoclax treatments were applied in this study to examine the safety doses and pharmacokinetics of this drug in humans. Several biomarkers have been identified such as pro-gastrin releasing peptide (pro-GRP) and an antibody marker for the cleaved product of caspase-mediated apoptosis (i.e., M30) to reflect the effect of navitoclax on BCL-2 protein expression and cell apoptosis respectively (Gandhi et al., 2011). The pro-GRP was known as a surrogate marker of BCL-2 amplification and its changes correlated with changes in tumor volume (Tahir et al., 2010; Gandhi et al., 2011). The study found that the severity of the major adverse events, including diarrhea, vomiting, fatigue and nausea were in grade 1 and 2, which were tolerable (Gandhi et al., 2011). Conversely, the blood platelet count was affected through different dosing regimens, whereby it lessened as the BCL-X_L_ mechanism was inhibited in response to elevation of the navitoclax concentration (Gandhi et al., 2011). Meanwhile, the best anti-tumor activity was observed in patients that were treated with the highest navitoclax dose (i.e., 130 mg). Furthermore, there was a correlation between pro-GRP levels with the best percentage tumor change and M30 levels also displayed a direct correlation with navitoclax doses. Hence, this study has established pro-GRP as a relevant biomarker to determine the BCL-2 level in response to its potential inhibitors.

Wilson et al. (2010) have conducted a phase 1 dose-escalation investigation on adult patients with various relapsed or refractory lymphoid tumors which were chronic lymphoid leukemia (CLL), diffuse large B-cell lymphoma (DLBCL), mantle-cell lymphoma (MCL), follicular lymphoma (FL), small lymphocytic lymphoma (SLL), classic Hodgkin’s lymphoma, NK/T-cell lymphoma and marginal-zone lymphoma. Intermittent and continuous dose-escalation regimens for 21 days had been carried out in this study and the patients were enrolled in different regimen groups through a modified Fibonacci 3 + 3 design (Wilson et al., 2010). Common toxic effects were observed throughout the treatment including gastrointestinal disorders, infection, fatigue thrombocytopenia, lymphocytopenia, and an increase in aminotransferases. The incidence of thrombocytopenia in patients receiving the intermittent dosing schedule was more severe than in the continuous regimens, whereby platelet counts dropped drastically with the first dose of each cycle, then followed by a slight rebound (Wilson et al., 2010). Nevertheless, the severity of thrombocytopenia was ameliorated by applying a continuous dosing schedule at day-8 of the treatment. They reported a novel pharmacodynamic effect of navitoclax on peripheral thrombocytopenia and T-cell lymphopenia in treating lymphoid tumors owing to high affinity inhibitions of BCL-2 and BCL-X_L_ proteins (Wilson et al., 2010). These results were in accordance with previous preclinical studies of navitoclax on SCLC and ALL xenograft models (Tse et al., 2008). Furthermore, they discovered that the hematological toxic effects were critical in patients with a limited bone marrow reserve (Wilson et al., 2010). Hence, Wilson et al. (2010) suggested starting the treatment with 150 mg of navitoclax for day 1–7, followed by a 325 mg dose administered on a continuous 21/21 dosing schedule for phase II studies (Wilson et al., 2010).

Roberts et al. (2012) have also carried out phase 1 clinical study of navitoclax, this time on 29 patients with relapsed or refractory CLL. A dose escalation regimen of navitoclax was implemented, where 15 patients received navitoclax for 14 days (10, 110, 200, 250 mg/d), whilst another 14 patients received navitoclax for 21 days (125, 200, 250, 200 mg/d) of each 21-days cycle (Roberts et al., 2012). However, the dose escalation was decided by continual reassessment methodology, in which this method can effectively estimate the maximum-tolerated dose (Roberts et al., 2012). Their study demonstrated that Navitoclax has significant single-agent activity against circulating, nodal, and splenic disease in patients with CLL. The anti-leukemic activity was seen within days, particularly in circulating lymphocyte counts, with maximum clinical responses typically observed within the first 4 months (Roberts et al., 2012). Thrombocytopenia due to BCL-X_L_ inhibition was observed in accordance with the results from preclinical studies of navitoclax with lymphocytic tumors. Hence, Roberts et al. (2012) suggested 250 mg/day of navitoclax in a continuous dosing regimen is effective for phase II studies.

#### Phase II Clinical Trials

A phase IIa clinical study of navitoclax has been conducted by Rudin et al. (2012) in patients with recurrent and progressive SCLC as the therapeutic choices for this occurrence is limited. They carried out a phase I study prior to the phase IIa to determine the optimum and safer navitoclax dose on patients with various solid tumors so that the risk of severe thrombocytopenia can be evaded. As a result, 325 mg daily dose was accepted to be used in phase II and it showed that the severity of thrombocytopenia can be managed (Rudin et al., 2012). Other than that, the effectiveness of cytotoxic drugs to kill the cancer cells in SCLC and other solid tumors could also be promoted in the presence of navitoclax (Rudin et al., 2012). Development of major adverse events in the Rudin et al. (2012) study corresponded to the Gandhi et al. (2011) report, which has been mentioned earlier. However, Rudin et al. (2012) observed around 40% occurrence of low blood platelet count in grade III-IV as the effect of navitoclax. Both studies suggested that combination therapy of navitoclax should be carried out to alleviate the adverse effects.

### Navitoclax Toxicity

Potential side effects have been discovered from the earlier study by Tse et al. (2008), where they reported a reversible thrombocytopenia with daily and escalation dose regimens on an animal model (i.e., dog). Even escalation dose regimen caused about 50% platelet drops initially, but a similar steady-state effect is induced through a continuous dosing at a constant high dosage (Tse et al., 2008). Similarly, transient thrombocytopenia and lymphopenia were observed as well by Shoemaker et al. (2008) and Lock et al. (2008) through their *in vivo* studies on SCLC and ALL xenograft models respectively. They have characterized this incident by rapid clearance of circulating platelets that are undergoing apoptosis in response to navitoclax treatment (Lock et al., 2008; Shoemaker et al., 2008; Tse et al., 2008). However, it is vital to note that the thrombocytopenia occurrence is reversible, well-tolerated and is mechanism-based. This outcome was supported by the evidence from its predecessor (i.e., ABT-737) data that showed an inactive ABT-737 enantiomer did not reduce the circulating platelet counts (Zhang et al., 2007). Fortunately, the absence of bone marrow toxicity was perceived in both ABT-737 and ABT-263 studies (Zhang et al., 2007; Tse et al., 2008). These results may lead to a potential clinical study of ABT-263 as a single agent or in a combination regime for SCLC and hematological malignancies treatment with tolerable side effects that could justify the existence of a therapeutic window with navitoclax treatment.

A very recent preclinical study has reported the establishment of navitoclax into a prodrug, namely a galacto-conjugation of navitoclax (Nav-Gal), which aims to minimize the platelet toxicity effect and increase the selectivity toward tumors-accumulating senescent cells (González‐Gualda et al., 2020). The modification of navitoclax with acetylated galactose exhibited selective apoptotic activity on senescent cells due to the elevation of lysosomal and galactosidase activity, which causes an active navitoclax to be released at the action site (González‐Gualda et al., 2020). However, this prodrug remained inactive in non-senescent cells, thus hindering the apoptotic mechanism of navitoclax. *In vitro* and *in vivo* studies of Nav-Gal on non-SCLC (NSCLC) cell lines and xenograft models have revealed a high potency of this prodrug to mitigate tumor progression (González‐Gualda et al., 2020). Besides, this novel prodrug did not trigger platelet apoptosis in mouse and human blood *ex vivo* (González‐Gualda et al., 2020). This reflects the capability of Nav-Gal to have an effective anti-tumor effect with the absence of thrombocytopenia which has been the highlight of their study. Further clinical studies of Nav-Gal on other cell lines and different xenograft models should be carried out to validate more the potential of this prodrug as an anti-cancer agent. Furthermore, it is worthwhile to conduct a clinical trial of Nav-Gal on NSCLC patients. This novel prodrug has given a promising outcome for navitoclax to exhibit its pro-apoptotic role with a high therapeutic index.

### The Association of B-Cell Lymphoma 2 Family Protein Expressions With Navitoclax Sensitivity

Most of the early studies on lung and lymphoid cancers that reported the effectiveness of navitoclax against BCL-2 family protein to induce cell death in SCLC and ALL xenograft models were associated with BCL-2 anti-apoptotic proteins’ expressions, including BCL-2, BCL-XL, BCL-W and MCL-1 (Lock et al., 2008; Shoemaker et al., 2008; Tse et al., 2008). Tumor cells with a high expression of MCL-1 create resistance toward navitoclax treatment. The low binding affinity of navitoclax toward MCL-1 (Tse et al., 2008) may contribute to this resistance as navitoclax would fail to block the interaction of MCL-1 with the executioner proteins, thus hindering cell death. Tse et al. (2008) have demonstrated that navitoclax potency was restored when MCL-1 expressions in several resistant cells were suppressed. They reported the sensitivity of SCLC and ALL xenograft models toward navitoclax treatment was positively correlated with BCL-2, BCL-XL, BCL-W and pro-apoptotic protein (i.e., BIM) expressions, while exhibiting a negative correlation with MCL-1 expression (Lock et al., 2008; Tse et al., 2008). In addition, Lock et al. (2008) have reported that the limited navitoclax activity on pediatric solid tumors was associated with a high level of MCL-1 expressions and one of the ALL xenograft models was not responsive to navitoclax due to lower expression of pro-apoptotic activators. These findings indicate a complex modulation of navitoclax efficacy by BCL-2 family protein expressions.

Various cancer types may exhibit different expression levels of this protein family which contribute to the varying potency of navitoclax. Many studies have demonstrated that the expression of BCL-2 family proteins is correlated with the navitoclax sensitivity at the cellular level for SCLC and ALL particularly. It is interesting to study the effect of a single navitoclax treatment on different cancer types with high BCL-2, BCL-XL or BCL-W expressions, and to include navitoclax in a combination treatment with other chemotherapeutic agents that have moderate anti-tumor action as well as tumor regression. Moreover, future studies can be done to determine whether the other types of lung cancers, lymphoid malignancies or other cancer types that are recently discovered to benefit from navitoclax treatment will display a similar or a new biomarker for navitoclax sensitivity. The results may allow us to evaluate the novel potential biomarkers for navitoclax.

## Role of Navitoclax in Fibrosis/Fibrotic Diseases

In wound healing condition, failure of myofibroblast to undergo apoptosis or convert back to the inactivated form of fibroblast would lead to excessive production of extracellular matrix (Latif et al., 2019), which contribute to the progression of fibrotic diseases (Ho et al., 2014). Uncontrolled activation of stiffness-induced myofibroblast results in pathological fibrosis (Kuehl and Lagares, 2018). Navitoclax has the ability to cause apoptosis of myofibroblast via a mitochondrial apoptosis mechanism. The apoptosis of myofibroblast may prevent and reverse the progression of fibrotic diseases such as in scleroderma, which is also known as systemic sclerosis (Wan Ali et al., 2015). Daily intake of navitoclax was shown to reverse dermal fibrosis via apoptosis of myofibroblast (Lagares et al., 2017). A summarization of navitoclax preclinical studies on different fibrotic diseases is displayed in Table 2.

**TABLE 2:** Summary of navitoclax action on fibrosis.

disease	Model	Dosage	Sample number	Side effect	Mechanism of action	References
Scleroderma dermal fibrosis	Mouse model	Given daily at 100 mg/kg	≥8 mice per group	None	Mitochondrial intrinsic apoptosis mechanism by inhibiting BCL-X_L_ in myofibroblast	Lagares et al. (2017)
Pulmonary fibrosis	Mouse model	50 mg/kg per day for 2 cycles (5 days per cycle), with 2 weeks gap between cycles	3 mice per group	None	Induce apoptosis by inhibiting BCL-X_L_ in senescent lung myofibroblast and senescent type II alveolar epithelial cells	Pan et al. (2017)
Biliary liver fibrosis	Mouse model and *in vitro* fibroblast culture	*Mdr* mice were given 50 mg/kg of navitoclax daily for 14 days while fibroblasts treated with 1 µM navitoclax for 24 h	3 mice per group	Neutropenia, thrombocytopenia	Inhibit BCL-X_L_ in senescent cholangiocytes and activated fibroblasts	Moncsek et al. (2018)
Cardiac fibrosis	Mouse model with either 13 weeks of age or 100 weeks of age	50 mg/kg per day for 2 cycles (7 days per cycle), with 1 week gap between cycles	4 – 15 per group	None	Induce apoptosis in aging cardiac cells and inhibits the expression of pro-fibrotic TGFβ2 protein	Walaszczyk et al. (2019)

BCL-X_L_, B-cell lymphoma-extra-large.

When a fibroblast is differentiated into a myofibroblast, the mitochondria are targeted by pro-apoptotic protein with the BH3 domain such as BIM (Lagares et al., 2017). In order to counteract the pro-apoptotic protein and to prevent the occurrence of apoptosis, the myofibroblasts express anti-apoptotic proteins such as BCL-X_L_. Navitoclax is a very potent BH3 mimetic drug that could inhibit BCL-X_L_ in myofibroblasts and eventually lead to the apoptosis of myofibroblasts (Lagares et al., 2017). Navitoclax acts on BCL-X_L_, sequestering BCL-X_L_ from binding with BIM. This enables BIM to continue functioning and causes apoptosis in rigid myofibroblasts. The inhibition of BCL-X_L_ by navitoclax has been shown to treat fibrosis in a mouse model of scleroderma dermal fibrosis via myofibroblast apoptosis. The main target of navitoclax in fibrosis-related-diseases is the antiapoptotic proteins of myofibroblast (Lagares et al., 2017). It has become clear that differentiated myofibroblast expresses an anti-apoptotic protein that inhibits the mechanism. By forcing these myofibroblasts to undergo apoptosis, the progression of fibrosis could be reversed (Kuehl and Lagares, 2018). Normally, fibroblasts would not be targeted by navitoclax as they are resistant to the action of navitoclax. However, stiffness-activated myofibroblast expresses anti-apoptotic proteins that sequester the function of BIM (Lagares et al., 2017). With the addition of navitoclax *in vivo*, anti-apoptotic BCL-X_L_ will be occupied by navitoclax and pro-apoptotic BIM will be set free to cause apoptosis. BIM will then bind to the BAX/BAK activator, resulting in mitochondria membrane permeabilization and apoptosis of myofibroblast. Therefore, navitoclax induces apoptosis of myofibroblast *in vivo* and treats dermal fibrosis by releasing pro-apoptotic BIM from BCL-X_L_ (Lagares et al., 2017).

In the case of persistent pulmonary fibrosis, navitoclax is potent in reversing the condition by killing senescent type II pneumocytes. A study conducted by Pan et al. (2017) where pulmonary fibrosis in mouse was induced through laser irradiation had found that the number of senescent cells increased significantly in pulmonary fibrosis. However, navitoclax as a senolytic drug killed the senescent cells and resolved pulmonary fibrosis (Pan et al., 2017). Likewise, navitoclax also exhibited an apoptotic effect on senescent lung myofibroblasts, which are the main effector cells in idiopathic pulmonary fibrosis (IPF) (Schafer et al., 2017). Continuous fibrosis in IPF is associated with the build-up of senescent type II alveolar epithelial cells and myofibroblasts that are resistant toward apoptosis. Navitoclax has been proven to treat permanent pulmonary fibrosis in mice through induction of apoptosis in senescent type II alveolar epithelial cells (Pan et al., 2017). Interestingly, navitoclax is shown to have both fibrinolytic and senolytic actions, which have a potential in reversing age-related fibrotic diseases. This drug acts as a selective, secure, and useful anti-fibrotic agent to reverse organ fibrosis (Kuehl and Lagares, 2018).

**FIGURE 4:**
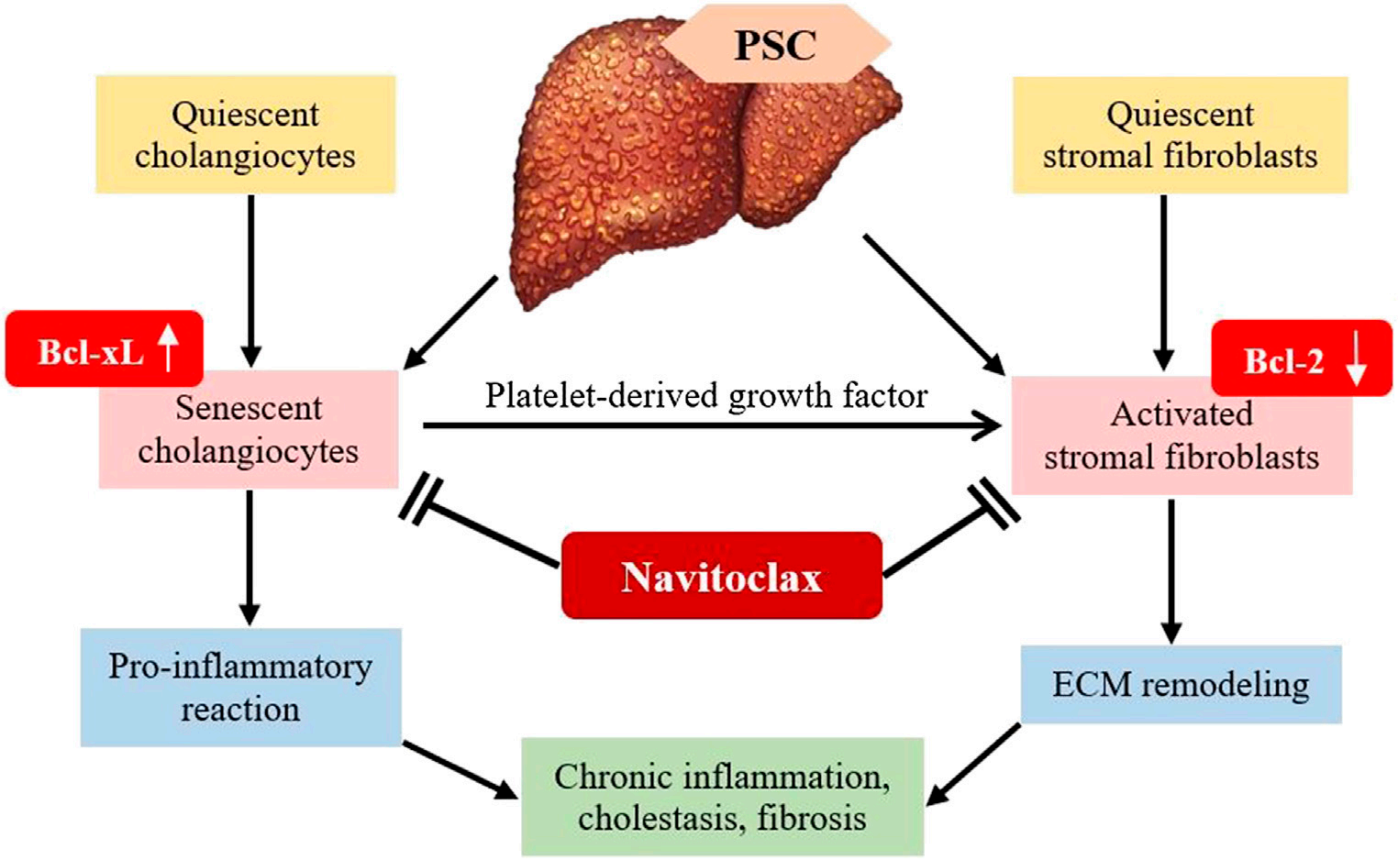
Mechanism of B-cell lymphoma-extra-large (BCL-X_L_) inhibitor in diminishing liver fibrosis. PSC, Primary sclerosing cholangitis; PDGF, Platelet-derived growth factor; ECM, extracellular matrix.

In the context of liver cirrhosis due to fibrosis, navitoclax specifically targets and binds to the inhibitory site of anti-apoptotic BCL-X_L_ protein. This will then stop the anti-apoptotic effect of BCL-X_L_, resulting in apoptosis of senescent cells (Zhu et al., 2016). The senolytic *in vivo* mechanism aids in diminishing liver fibrosis via the apoptosis of senescent liver cells (Moncsek et al., 2018). Primary sclerosing cholangitis (PSC) is usually associated with senescence of cholangiocyte (Moncsek et al., 2018). As shown in Figure 4, PSC continuous injury to cholangiocytes causes a pro-inflammatory response, which induces chronic inflammation, cholestasis and fibrosis. The majority of the cholangiocytes turn into senescent cells and express BCL-X_L_ to prevent death. Senescent cholangiocytes secrete growth factors such as platelet-derived growth factor (PDGF) that activate stromal fibroblasts. Continual activation of fibroblast is the key factor of liver fibrosis. Activated stromal fibroblasts express a lower level of BCL-2, causing them to become BCL-X_L_-dependent. Since BCL-X_L_ is the key to survival of senescent cholangiocytes and activated stromal fibroblasts, treatment with navitoclax, which is a BCL-X_L_ inhibitor is potent in reducing biliary liver fibrosis (Moncsek et al., 2018).

Senescent cholangiocytes consistently secrete growth factors that stimulate stromal fibroblasts, which contribute to the progression of fibrosis (Karin et al., 2016). The activated stromal fibroblasts show increased responsiveness toward apoptotic signals (Mertens et al., 2013). Moncsek et al. (2018) operated a coculture system and identified that senescent cholangiocytes activate mesenchymal cells in the presence of PDGF into stromal fibroblast. The key to the survival of PDGF-induced human and mouse fibroblasts is BCL-X_L_. Moreover, BCL-X_L_ is also expressed in senescent cholangiocytes. Thus, the blockage of BCL-X_L_ by navitoclax promotes apoptosis in PDGF-activated fibroblasts and senescent cholangiocytes *in vitro*. Moncsek et al. (2018) examined navitoclax efficacy on both activated stromal fibroblasts and senescent cholangiocytes in the multidrug resistance 2 gene knockout (*Mdr*2^−/−^) mouse model of biliary liver fibrosis. The reduction of senescent cholangiocytes and activated stromal fibroblasts due to apoptosis reversed established biliary liver fibrosis in *Mdr2* ^*−/−*^ mice.

According to Walaszczyk et al. (2019), aging cardiomyocytes contribute the most to defective cardiac function as they are abundant in cardiac tissues, which subsequently lead to cardiac fibrosis. This can lead to a higher chance of mortality in the occurrence of myocardial infarction (MI). Cardiac fibrosis due to aging cells results in reduced survival of the elderly with MI. The treatment of aged mice with navitoclax successfully induces apoptosis in aging cardiac cells and inhibits the expression of pro-fibrotic transforming growth factor beta-2 (TGFβ2) protein by aged mice, thereby decreasing cardiac fibrosis. Particularly, the removal of aging cardiac cells ameliorated myocardial remodeling and diastolic activity along with a higher rate of survival in the event of MI.

Since both senescent cells and activated myofibroblasts express BCL-X_L_ proteins, navitoclax can induce apoptosis not only in myofibroblasts, but also in senescent cells. The specificity of navitoclax enables the eradication of both myofibroblasts and related senescent cells, hence resolving fibrosis in the affected organs. This widens the potential of navitoclax in treating a variety of fibrotic diseases.

## Navitoclax in Combination Therapy

To date, most studies on navitoclax with the presence of other drugs have been conducted against various solid tumors and lymphoid malignancies. Researches intend to amplify the anti-tumor activity of chemotherapeutic drugs with navitoclax through multiple simultaneous protein interactions. This could be achieved as the chemotherapeutic agents commonly display distinct target proteins from navitoclax. The preclinical and clinical studies on navitoclax in combination treatment are summarized in Table 3.

**TABLE 3:** Summary of navitoclax combination effect with other drugs.

disease	Model	Combination drug	Advantages/Disadvantages	References
PTC	K1 human BRAF^V600E^-positive PTC cell line	Navitoclax + vemurafenib	Induce apoptosis by inhibiting p-Erk 1/2, BCL-X_L_ and BCL-2	Jeong et al. (2019)
NSCLC	NSCLC cell line	Benzimidazoles + navitoclax	Enhance cancer cells apoptosis by upregulating NOXA protein expression	Lam et al. (2015)
SCLC	H69, H526, H82, H209, H2171 and H2141 human SCLC cell lines	Vorinostat + navitoclax	Induce cell death via upregulation of NOXA or BIM and downregulation of BCL-X_L_	Nakajima et al. (2016)
AML	Bone marrow cells from NUP98-NSD1^+^/FLT3-ITD^+^ and NUP98-NSD1^-^/FLT3-ITD^+^ AML patients	Navitoclax + dasatinib	Induce cell death by inhibiting BCL-2A1, proteins lck and fgr	Kivioja et al. (2019)
Chronic lymphocytic leukemia (CLL)	Human model – random patients	Navitoclax + rituximab	Potentiates anti-leukemic effect of rituximab alone	Kipps et al. (2015)
B-cell lymphoid cancer	Human model – 29 patients	Navitoclax + rituximab	Overcome the resistance problem against a range of chemotherapeutic drug	Roberts et al. (2015)
NHL	Mouse xenograft model of NHL	Navitoclax + bendamustine ± rituximab	Inhibition of tumor growth	Ackler et al. (2012)
Advanced solid tumors	Human model – 11 patients	Navitoclax + erlotinib	No efficacy observed with the presence of side effects	Tolcher et al. (2015)
Ovarian cancer	Ovcar-3, Ovcar-4, Ovcar-5, Ovcar-8, Igrov-1, Skov-3 cell lines	Navitoclax + carboplatin + paclitaxel	Retards cancer cell proliferation by upregulating caspase3/7	Stamelos et al. (2013)

BCL‐XL, B‐cell lymphoma‐extra‐large; PTD, Papillary thyroid cancer; NSCLC, Non‐small cell lung cancer; SCLC, Small cell lung cancer; AML, Acute myeloid leukemia; NHL, Non‐Hdgkin's lymphoma.

BRAF^V600E^ mutation in papillary thyroid cancer (PTC) highly corresponds to aggressive tumor features, including metastasis, cancer relapse, and failure of radioiodine treatment (Zhu et al., 2019). Vemurafenib, a potent BRAF inhibitor that exhibited strong efficiency in metastatic malignant melanoma possessing BRAF^V600E^ mutation (Mcarthur et al., 2014), also showed a convincing therapeutic effect against BRAFV600E-positive PTC (Kim et al., 2013; Brose et al., 2016). Nevertheless, the development of resistance toward vemurafenib slowly limited its efficacy in BRAFV600E-positive PTC (Dadu et al., 2015). In a study carried out by Jeong et al. (2019), vemurafenib alone demonstrated anti-proliferative activity against K1 BRAFV600E-positive PTC cells by suppressing almost half of K1 cells growth at 10 µM. After the treatment of vemurafenib for 24 h, the protein expression of p-Erk1/2 was reduced, while the protein expression of BCL-X_L_ and BCL-2 was increased. This proved that vemurafenib upregulates the expression of anti-apoptotic BCL-2 and BCL-X_L_ in K1 cells. On the other hand, treatment of navitoclax alone at 4 µM for 24 h showed insignificant outcomes on the survival of K1 cells. Therefore, a combination of navitoclax and vemurafenib was given against K1 BRAFV600E-positive PTC cells. This combination strongly inhibited cell development and induced a higher rate of apoptosis with a lower concentration of navitoclax and vemurafenib, in which 0.5 and 1 µM were required respectively to yield synergistic activity (Jeong et al., 2019).

A study conducted by Lam et al. (2015) proved that a combination of benzimidazoles, an anti-helminthic agent with navitoclax enhanced the apoptosis effect in NSCLC cell lines via a mechanism mediated by NOXA, a pro-apoptotic protein with BH3 domain. The addition of benzimidazoles upregulates both mRNA and protein expression of pro-apoptotic NOXA, which then binds and opposes the effect of anti-apoptotic protein MCL-1. This combination is beneficial as navitoclax alone is unable to target and bind on MCL-1 due to its low affinity to MCL-1 (Leverson et al., 2015). Combination therapy of navitoclax with other agents that target MCL-1 could restore the apoptosis process in cancers with high expression of BCL-X_L_ and MCL-1 (Lam et al., 2015).

The anti-apoptotic BCL-X_L_ and MCL-1 are highly expressed in SCLC, as SCLC relies on both BCL-X_L_ and MCL-1 for survival (Nakajima et al., 2016). Nakajima et al. (2016) reported that a combination of navitoclax and vorinostat, which is known as a histone deacetylase (HDAC) inhibitor, effectively triggered apoptosis in various SCLC cell lines, including navitoclax-resistant H82 and H526 cells. This is based on the mechanism that NOXA and BIM can be transcriptionally induced by HDAC inhibitors (Nakajima et al., 2016) as histone modification plays a significant role in SCLC (Peifer et al., 2012). Therefore, vorinostat as a HDAC inhibitor is capable of mutating histone modification, which then augments SCLC cell death by navitoclax due to the expression of NOXA and BIM. SCLC has increased responsiveness toward navitoclax with the increased expression of NOXA and BIM, where pro-apoptotic NOXA binds to anti-apoptotic MCL-1 with a high affinity that leads to degeneration of MCL-1 (Nakajima et al., 2016). NOXA was constantly activated by the combination of vorinostat and navitoclax in all tested cell lines excluding H209 cells, which do not have known levels of NOXA and BIM. However, H209 cells were effectively killed when vorinostat and navitoclax co-treatment was given. This was achieved via the decreased protein expression of BCL-X_L_ induced only by the combination of vorinostat and navitoclax, causing the discharge of BAK from BCL-X_L_ into an active form. Hence, Nakajima et al. (2016) proved that the combination treatment significantly activated apoptosis in certain cell lines by upregulating NOXA or BIM while inhibiting BCL-X_L_ alone in other cell lines.

Acute myeloid leukemia (AML) with co-existing NUP98-NSD1 and FLT3-ITD mutation is accompanied by poor prognosis and a low chance of survival (Akiki et al., 2010; Hollink et al., 2011; Ostronoff et al., 2014; Thanasopoulou et al., 2014). Kivioja et al. (2019) discovered that a combination of Src/Abl-inhibitor dasatinib and BCL-2 blocker navitoclax works synergistically against NUP98-NSD1^+^/FLT3-ITD^+^ AML cells. Patient cells with NUP98-NSD1^+^/FLT3-ITD^+^ were very responsive toward navitoclax and showed the most sensitivity toward dasatinib. There was an increased expression of BCL-2A1, proteins Lck and Fgr in NUP98-NSD1^+^/FLT3-ITD^+^ AML cells. On the other hand, the expression of BCL-2A1, proteins Lck and Fgr were much lower in healthy CD34 ^+^ cells, as proven by gene expression profiling. Therefore, a combined treatment of navitoclax and dasatinib produces synergistic outcomes against AML cells co-expressing NUP98-NSD1+ and FLT3-ITD+ in which navitoclax inhibits BCL-2A1 while dasatinib inhibits proteins Lck and Fgr (Kivioja et al., 2019).

Navitoclax alone adequately exhibits therapeutic response against CLL and lymphoma as described above. Kipps et al. (2015) carried out a study using a combination of navitoclax and rituximab for CLL patients. The combination regimen was given to a group of random patients once a week, for 8 weeks. Rituximab alone was given to another group of CLL patients once a week for the same duration. The combination of navitoclax plus rituximab was shown to give a better therapeutic effect compared to rituximab alone. CLL cells express a high level of anti-apoptotic protein BCL-2 (Fegan and Pepper, 2013), which can be easily targeted and inhibited by navitoclax. At the same time, CLL cells also express a high level of pro-apoptotic protein BIM, causing the CLL cells to be more susceptible to apoptosis with navitoclax treatment. The pro-apoptotic protein BIM will be displaced and set free by navitoclax to carry out the apoptotic program of CLL cells (Kipps et al., 2015). Therefore, navitoclax potentiates the anti-leukemic effect of rituximab. CLL patients treated with a combination of navitoclax and rituximab showed a higher overall response rate compared to those treated with rituximab alone (Kipps et al., 2015).

Navitoclax and rituximab produce a synergistic effect in pre-clinical models of B-cell lymphoid cancers (Roberts et al., 2015). *In vitro*, lymphoma clones with resistance to rituximab show elevated expression of anti-apoptotic BCL-2 family proteins and increased level of resistance to a range of chemotherapeutic drugs. These findings suggest that by reducing the apoptotic threshold, navitoclax as a BCL-2 inhibitor may produce a synergistic effect with rituximab against B-cell malignancies (Roberts et al., 2015). In the experiment designed by Roberts et al. (2015), 29 patients were administered a 200–325 mg daily dose of navitoclax and four weekly doses of rituximab. The outcome of the combination was well-tolerated, with a few side effects including mild diarrhea, nausea and thrombocytopenia. The total CD19^+^ was significantly decreased, while CD2^+^ cells and serum IgM were also decreased in the first year. The ultimate acceptable dose for navitoclax in combination with rituximab was 250 mg/dose (Roberts et al., 2015), with no interaction between the drugs. This combination exhibited greater efficacy for low-grade lymphoid cancers than either navitoclax or rituximab alone (Roberts et al., 2015).

An *in vivo* research done by Ackler et al. (2012) showed that the therapeutic combinations of navitoclax with either bendamustine alone or bendamustine with rituximab (BR) were effective in mouse xenograft models of non-Hodgkin’s lymphoma (NHL). These combinations were applied in a few models of NHL, including the DoHH-2 DLBCL model, Granta 519 MCL model and RAMOS BL model. Navitoclax enhanced the effect of bendamustine in every cell line experimented. Bendamustine stimulated p53 in Granta 519 tumors and activated caspase 3 at the same time. Navitoclax also enhanced the reaction of a subset of tumors to bendamustine-rituximab (BR). The addition of navitoclax essentially boosted the durability and extent of tumor response to bendamustine and BR, resulting in inhibition of tumor growth (Ackler et al., 2012).

Pre-clinically, navitoclax has been shown to yield an *in vitro* synergistic outcome with erlotinib, an epidermal growth factor receptor inhibitor. Erlotinib inhibits the anti-apoptotic MCL-1 protein and activates the pro-apoptotic BIM protein (Chen et al., 2011). Tolcher et al. (2015) tested a combination of navitoclax and erlotinib in an *in vivo* phase I study. Eleven patients with known cancers undergoing erlotinib treatment were given 150 mg erlotinib in addition to 150 mg navitoclax orally once per day. The pharmacokinetic evaluation presented no possible interactions between co-administered navitoclax and erlotinib. Although the maximum tolerated dose of navitoclax was given at 150 mg/d, the experiment did not achieve the desired outcome with 0% response rate for the navitoclax and erlotinib combination. Moreover, a few side effects can be observed, such as diarrhea, nausea and vomiting.

Carboplatin and paclitaxel are the most prevalent chemotherapeutic drugs used in ovarian cancer therapy. However, the evolution of drug resistance mechanism allows long term survival of cancer cells. This required the use of navitoclax to overcome the resistance of ovarian cancer cells toward carboplatin and paclitaxel (Wong et al., 2012). In the *in vitro* treatment of ovarian cancer done by Stamelos et al. (2013), the combination of navitoclax with either carboplatin or paclitaxel retarded cancer cell proliferation significantly as compared to the combination of carboplatin and paclitaxel, which acted antagonistically to one another in Ovcar-4, Ovcar-8 and Skov-3 cells. When navitoclax was combined with carboplatin and paclitaxel, the antagonism between carboplatin and paclitaxel was diminished. The synergistic effect was produced from the combination therapy in comparison to either carboplatin or paclitaxel monotherapy. Furthermore, navitoclax strengthened the effect of carboplatin and paclitaxel by upregulating caspase3/7 in Igrov-1 spheroids, thus suppressing the proliferation of Igrov-1. The triplet combination of navitoclax, carboplatin and paclitaxel presented beyond additive effect toward Igrov-1 spheroids (Stamelos et al., 2013).

The role of navitoclax can be major or auxiliary, depending on the underlying cause of the disease. This is because different diseases have different mutations, which can be inhibited by certain chemotherapeutic drugs. In most diseases, navitoclax functions as an auxiliary drug. The auxiliary effect of navitoclax can be seen when certain diseases have developed resistance against the specific chemotherapeutic drugs, or the treatment of specific chemotherapeutic drugs upregulates the expression of BCL-XL proteins. On the contrary, there are certain cases where navitoclax acts as a major drug in treating diseases that are caused by the overexpression of anti-apoptotic BCL2, or BCL-X_L_ proteins. However, in diseases such as SCLC and AML, navitoclax plays neither the auxiliary nor major role, but instead acts as a co-inhibitor with other drugs to inhibit targeted proteins respectively, thus resulting in synergistic therapeutic outcomes.

## Conclusion and Future Prospects

The BCL-2 family of proteins is largely identified in normal cells and plays a crucial role in cell death and survival. Its anti-apoptotic role and overexpression in cancer cells and fibroblasts have been widely studied and identified as a valid and potential target for anti-cancer and anti-fibrotic therapies. Navitoclax has now been in clinical studies for SCLC treatment, though, the data of navitoclax on various solid tumor types are still limited. More studies should be done to explore and target the molecular mechanism that associates with navitoclax’s effect in solid tumors and hematologic tumors (except for ALL). Elucidation of this interaction would assist in determining the potential of navitoclax as a single agent or can be used in combinational regimens for these cancers' treatment. Other than that, following Lee et al. (2018) study, a further *in vivo* study of navitoclax against several breast cancer xenograft models should be done to determine its efficacy and to find out any complications. Animal models such as dogs or pigs could be used in the *in vivo* study before pursuing human clinical trials in order to get a more accurate and valid preclinical data. Moreover, it is worth pursuing the development of navitoclax as an anti-oral-cancer drug as Yang et al. (2019) have obtained a promising result with a novel molecular target. Advanced mechanism-based research on navitoclax and with other therapeutic agents should be pursued to achieve greater success at the pre-clinical stage, then translated into potential clinical therapies.

Most of the previous, as well as ongoing clinical studies on navitoclax are focusing more on its therapeutic effect as a pro-apoptotic agent on cancer and tumor therapies. However, cell apoptotic activity mediated by navitoclax may also provide a therapeutic outcome on cardiovascular diseases. Some of the cardiovascular diseases such as atherosclerosis, myocardial infarction, and stroke are exacerbated due to excessive cellular proliferation. On that account, pro-apoptotic agents like navitoclax may play a role in ameliorating the progression of those diseases. In order to approach this matter, *in vitro* studies of navitoclax on various primary cell lines (i.e., endothelial cells) has to be done in order to evaluate the potency of navitoclax.

Based on the evidence discussed in this review paper, the navitoclax mechanism is shown to be complex and diverse. Through SCLC and several lymphoid malignancies, navitoclax can be well-explained to have an effect through the inhibition of either BCL-2, BCL-W or BCL-X_L_ activities, with MCL-1 expression having minor influence However, further downstream signaling of the intrinsic apoptotic pathway through BCL-2 family members is modulated by the expression of proapoptotic activators such as BIM, and executioner proteins as well. This downstream signaling has contributed to the complex mechanism of navitoclax as those proteins’ expressions should be considered in evaluating the effectiveness of single navitoclax treatments. Otherwise, a combination of navitoclax with other cytotoxic agents would be the best strategy to enhance its anti-tumor effect. Most of the combination treatment clinical studies have also shown a tremendous therapeutic effect of navitoclax as a proapoptotic agent with very minimal unfavorable effects. Furthermore, accumulating findings on solid tumors have uncovered several novel molecular mechanisms targeted by navitoclax to induce cell killing activity such as survivin and CHOP. These data suggest that navitoclax has a diverse mechanism with multiple molecular targets that are not only limited through BCL-2 family members in order to exhibit its role as a pro-apoptotic agent. Therefore, clear elucidation and understanding of potential molecular targets by navitoclax is essential and highly beneficial for further development of navitoclax treatment in cancer as well as other diseases including cardiovascular disease.

## Author Contributions

NSNH and LSL searched the literature and drafted the manuscript. AU and NMA edited and revised the manuscript. All authors approved the final version of the manuscript.

## Funding

This review was supported by a grant from Ministry of Education, Malaysia (FRGS/1/2019/SKK06/UKM/02/7) and Universiti Kebangsaan Malaysia (UKM).

## Conflict of Interest

The authors declare that the research was conducted in the absence of any commercial or financial relationships that could be construed as a potential conflict of interest.

## References

[B1] Ackler S. Mitten M. J. Chen J. Clarin J. Foster K. Jin S. (2012). Navitoclax (ABT-263) and bendamustine ± rituximab induce enhanced killing of non-Hodgkin's lymphoma tumoursin vivo. Br. J. Pharmacol. 167, 881–891. 10.1111/j.1476-5381.2012.02048.x 22624727 PMC3575786

[B2] Ackler S. Mitten M. Chen J. Clarin J. Foster K. Jin S. (2012b). Navitoclax (ABT-263) and bendamustine ± rituximab induce enhanced killing of non-Hodgkin’s lymphoma tumoursin *vivo* . Br. J. Pharmacol. 167, 881–891. 10.1111/j.1476-5381.2012.02048.x 22624727 PMC3575786

[B3] Ackler S. Mitten M. J. Foster K. Oleksijew A. Refici M. Tahir S. K. (2010). The Bcl-2 inhibitor ABT-263 enhances the response of multiple chemotherapeutic regimens in hematologic tumors *in vivo* . Cancer Chemother. Pharmacol. 66, 869–880. 10.1007/s00280-009-1232-1 20099064

[B4] Akiki S. Dyer S. A. Grimwade D. Ivey A. Abou-Zeid N. Borrow J. NUP98-NSD1fusion in association withFLT3-ITD mutation identifies a prognostically relevant subgroup of pediatric acute myeloid leukemia patients suitable for monitoring by real time quantitative PCR. Genes Chromosomes Cancer 52, 1053–1064. 10.1002/gcc.22100 23999921

[B5] Bender T. Martinou J.-C. (2013). Where killers meet--permeabilization of the outer mitochondrial membrane during apoptosis. Cold Spring Harb. Perspect. Biol. 5, a011106 10.1101/cshperspect.a011106 23284044 PMC3579396

[B6] Brose M. S. Cabanillas M. E. Cohen E. E. W. Wirth L. J. Riehl T. Yue H. (2016). Vemurafenib in patients with BRAFV600E-positive metastatic or unresectable papillary thyroid cancer refractory to radioactive iodine: a non-randomised, multicentre, open-label, phase 2 trial. Lancet Oncol. 17, 1272–1282. 10.1016/s1470-2045(16)30166-8 27460442 PMC5532535

[B7] Bruncko M. Oost T. K. Belli B. A. Ding H. Joseph M. K. Kunzer A. (2007). Studies leading to potent, dual inhibitors of Bcl-2 and Bcl-xL. J. Med. Chem. 50, 641–662. 10.1021/jm061152t 17256834

[B8] Chen J. Jin S. Abraham V. Huang X. Liu B. Mitten M. J. (2011). The Bcl-2/Bcl-XL/Bcl-w inhibitor, navitoclax, enhances the activity of chemotherapeutic agents *in vitro* and *in vivo* . Mol. Cancer Ther. 10, 2340–2349. 10.1158/1535-7163.mct-11-0415 21914853

[B9] Chen L. Willis S. N. Wei A. Smith B. J. Fletcher J. I. Hinds M. G. (2005). Differential targeting of prosurvival Bcl-2 proteins by their BH3-only ligands allows complementary apoptotic function. Mol. Cell 17, 393–403. 10.1016/j.molcel.2004.12.030 15694340

[B10] Dadu R. Shah K. Busaidy N. L. Waguespack S. G. Habra M. A. Ying A. K. (2015). Efficacy and tolerability of vemurafenib in patients with BRAFV600E -positive papillary thyroid cancer: M.D. Anderson cancer center off label experience. J. Clin. Endocrinol. Metabol. 100, E77–E81. 10.1210/jc.2014-2246 PMC4283003 25353071

[B11] Fegan C. Pepper C. (2013). “Apoptosis deregulation in CLL,” in Advances in chronic lymphocytic leukemia Cardiff, Wales, UK:(Springer), 151–171. 10.1007/978-1-4614-8051-8_7 24014296

[B12] Gandhi L. Camidge D. R. Ribeiro de Oliveira M. Bonomi P. Gandara D. Khaira D. (2011). Phase I study of Navitoclax (ABT-263), a novel Bcl-2 family inhibitor, in patients with small-cell lung cancer and other solid tumors. J. Clin. Oncol. 29, 909–916. 10.1200/jco.2010.31.6208 21282543 PMC4668282

[B13] Gazitt Y. Fey V. Thomas C. Alvarez R. (1998). Bcl-2 overexpression is associated with resistance to dexamethasone, but not melphalan, in multiple myeloma cells. Int. J. Oncol. 13, 397–405. 10.3892/ijo.13.2.397 9664139

[B14] González-Gualda E. Pàez-Ribes M. Lozano-Torres B. Macias D. Wilson J. R. González-López C. (2020). Galacto‐conjugation of Navitoclax as an efficient strategy to increase senolytic specificity and reduce platelet toxicity. Aging Cell 19 (4), e13142 10.1111/acel.13142 32233024 PMC7189993

[B15] Han Z. Liang J. Li Y. He J. (2019). Drugs and clinical approaches targeting the antiapoptotic protein: a review. Biomed. Res. Int. 2019, 1212369 10.1155/2019/1212369 31662966 PMC6791192

[B16] Ho Y. Y. Lagares D. Tager A. M. Kapoor M. (2014). Fibrosis-a lethal component of systemic sclerosis. Nat. Rev. Rheumatol. 10, 390 10.1038/nrrheum.2014.53 24752182

[B17] Hollink I. H. I. M. Van Den Heuvel-Eibrink M. M. Arentsen-Peters S. T. C. J. M. Pratcorona M. Abbas S. Kuipers J. E. (2011). NUP98/NSD1 characterizes a novel poor prognostic group in acute myeloid leukemia with a distinct HOX gene expression pattern. Blood 118, 3645–3656. 10.1182/blood-2011-04-346643 21813447

[B18] Ibrahim N. Nazimi A. J. Ajura A. J. Nordin R. Latiff Z. A. Ramli R. (2016). The clinical features and expression of bcl-2, cyclin D1, p53, and proliferating cell nuclear antigen in syndromic and nonsyndromic keratocystic odontogenic tumor. J. Craniofac. Surg. 27, 1361–1366. 10.1097/scs.0000000000002792 27391504

[B19] Jeong J. H. Oh J. M. Jeong S. Y. Lee S.-W. Lee J. Ahn B.-C. (2019). Combination treatment with the BRAFV600E inhibitor vemurafenib and the BH3 mimetic navitoclax for BRAF-mutant thyroid carcinoma. Thyroid 29, 540–548. 10.1089/thy.2018.0511 30869573

[B20] Jha K. Shukla M. Pandey M. (2012). Survivin expression and targeting in breast cancer. Surg. Oncol. 21, 125–131. 10.1016/j.suronc.2011.01.001 21334875

[B21] Karin D. Koyama Y. Brenner D. Kisseleva T. (2016). The characteristics of activated portal fibroblasts/myofibroblasts in liver fibrosis. Differentiation 92, 84–92. 10.1016/j.diff.2016.07.001 27591095 PMC5079826

[B22] Kawatani M. Imoto M. (2003). Deletion of the BH1 domain of Bcl-2 accelerates apoptosis by acting in a dominant negative fashion. J. Biol. Chem. 278, 19732–19742. 10.1074/jbc.m213038200 12644466

[B23] Kim K. B. Cabanillas M. E. Lazar A. J. Williams M. D. Sanders D. L. Ilagan J. L. (2013). Clinical responses to vemurafenib in patients with metastatic papillary thyroid cancer harboring BRAFV600E mutation. Thyroid 23, 1277–1283. 10.1089/thy.2013.0057 23489023 PMC3967415

[B24] Kipps T. J. Eradat H. Grosicki S. Catalano J. Cosolo W. Dyagil I. S. (2015). A phase 2 study of the BH3 mimetic BCL2 inhibitor navitoclax (ABT-263) with or without rituximab, in previously untreated B-cell chronic lymphocytic leukemia. Leuk. Lymphoma 56, 2826–2833. 10.3109/10428194.2015.1030638 25797560 PMC4643417

[B25] Kivioja J. L. Thanasopoulou A. Kumar A. Kontro M. Yadav B. Majumder M. M. (2019). Dasatinib and navitoclax act synergistically to target NUP98-NSD1+/FLT3-ITD+ acute myeloid leukemia. Leukemia 33, 1360–1372. 10.1038/s41375-018-0327-2 30568173

[B26] Kogan A. J. Haren M. (2008). Translating cancer trial endpoints into the language of managed care. Biotechnol. Healthc. 5, 22–35. 22478698 PMC2651701

[B27] Kuehl T. Lagares D. (2018). BH3 mimetics as anti-fibrotic therapy: unleashing the mitochondrial pathway of apoptosis in myofibroblasts. Matrix Biol. 68–69, 94–105. 10.1016/j.matbio.2018.01.020 29408011

[B28] Lagares D. Santos A. Grasberger P. E. Liu F. Probst C. K. Rahimi R. A. (2017). Targeted apoptosis of myofibroblasts with the BH3 mimetic ABT-263 reverses established fibrosis. Sci. Transl. Med. 9, eaal3765 10.1126/scitranslmed.aal3765 29237758 PMC8520471

[B29] Lam L. T. Zhang H. Xue J. Leverson J. D. Bhathena A. (2015). Antihelminthic benzimidazoles potentiate navitoclax (ABT-263) activity by inducing Noxa-dependent apoptosis in non-small cell lung cancer (NSCLC) cell lines. Cancer Cell Int. 15, 5 10.1186/s12935-014-0151-3 25685063 PMC4326508

[B30] Latif M. A. Ibrahim F. W. Arshad S. A. Hui C. K. Jufri N. F. Hamid A. (2019). Cytotoxicity, proliferation and migration rate assessments of human dermal fibroblast adult cells using Zingiber Zerumbet extract. Sains Malays. 48, 121–127. 10.17576/jsm-2019-4801-14

[B31] Lee E. Y. Gong E.-Y. Shin J.-S. Moon J.-H. Shim H. J. Kim S.-M. (2018). Human breast cancer cells display different sensitivities to ABT-263 based on the level of survivin. Toxicol. Vitro 46, 229–236. 10.1016/j.tiv.2017.09.023 28947240

[B32] Leverson J. Zhang H. Chen J. Tahir S. Phillips D. Xue J. (2015). Potent and selective small-molecule MCL-1 inhibitors demonstrate on-target cancer cell killing activity as single agents and in combination with ABT-263 (navitoclax). Cell Death Dis. 6, e1590 10.1038/cddis.2014.561 25590800 PMC4669759

[B33] Liu J. Nie Z. Lei Y. Yang S. Liu Z. (2017). The expression of ERβ2, Bcl-xl and Bax in non-small cell lung cancer and associated with prognosis. Int. J. Clin. Exp. Pathol. 10, 10040–10046. 10.1177/0300060519871373 31966894 PMC6965973

[B34] Lock R. Carol H. Houghton P. J. Morton C. L. Kolb E. A. Gorlick R. (2008). Initial testing (stage 1) of the BH3 mimetic ABT-263 by the pediatric preclinical testing program. Pediatr. Blood Cancer 50, 1181–1189. 10.1002/pbc.21433 18085673

[B35] Lv Y. G. Yu F. Yao Q. Chen J. H. Wang L. (2010). The role of survivin in diagnosis, prognosis and treatment of breast cancer. J. Thorac. Dis. 2, 100–110. 22263027 PMC3256445

[B36] Mcarthur G. A. Chapman P. B. Robert C. Larkin J. Haanen J. B. Dummer R. (2014). Safety and efficacy of vemurafenib in BRAFV600E and BRAFV600K mutation-positive melanoma (BRIM-3): extended follow-up of a phase 3, randomised, open-label study. Lancet Oncol. 15, 323–332. 10.1016/s1470-2045(14)70012-9 24508103 PMC4382632

[B37] Mérino D. Khaw S. L. Glaser S. P. Anderson D. J. Belmont L. D. Wong C. (2012). Bcl-2, Bcl-xL, and Bcl-w are not equivalent targets of ABT-737 and navitoclax (ABT-263) in lymphoid and leukemic cells. Blood 119, 5807–5816. 10.1182/blood-2011-12-400929 22538851 PMC3382939

[B38] Mertens J. C. Fingas C. D. Christensen J. D. Smoot R. L. Bronk S. F. Werneburg N. W. (2013). Therapeutic effects of deleting cancer-associated fibroblasts in cholangiocarcinoma. Cancer Res. 73, 897–907. 10.1158/0008-5472.can-12-2130 23221385 PMC3549008

[B39] Moldoveanu T. Follis A. V. Kriwacki R. W. Green D. R. (2014). Many players in BCL-2 family affairs. Trends Biochem. Sci. 39, 101–111. 10.1016/j.tibs.2013.12.006 24503222 PMC4005919

[B40] Monaco G. Decrock E. Nuyts K. Wagner L. E. Ii Luyten T. Strelkov S. V. (2013). Alpha-helical destabilization of the Bcl-2-BH4-domain peptide abolishes its ability to inhibit the IP3 receptor. PLoS One 8, e73386 10.1371/journal.pone.0073386 24137498 PMC3795776

[B41] Moncsek A. Al-Suraih M. S. Trussoni C. E. O'hara S. P. Splinter P. L. Zuber C. (2018). Targeting senescent cholangiocytes and activated fibroblasts with B-cell lymphoma-extra large inhibitors ameliorates fibrosis in multidrug resistance 2 gene knockout (Mdr2−/−ar mice. Hepatology 67, 247–259. 10.1002/hep.29464 28802066 PMC5739965

[B42] Nakajima W. Sharma K. Hicks M. A. Le N. Brown R. Krystal G. W. (2016). Combination with vorinostat overcomes ABT-263 (navitoclax) resistance of small cell lung cancer. Cancer Biol. Ther. 17, 27–35. 10.1080/15384047.2015.1108485 26575826 PMC4847809

[B43] Oakes S. R. Vaillant F. Lim E. Lee L. Breslin K. Feleppa F. (2012). Sensitization of BCL-2-expressing breast tumors to chemotherapy by the BH3 mimetic ABT-737. Proc. Natl. Acad. Sci. U.S.A. 109, 2766–2771. 10.1073/pnas.1104778108 21768359 PMC3286989

[B44] Ostronoff F. Othus M. Gerbing R. B. Loken M. R. Raimondi S. C. Hirsch B. A. (2014). NUP98/NSD1 and FLT3/ITD coexpression is more prevalent in younger AML patients and leads to induction failure: a COG and SWOG report. Blood 124, 2400–2407. 10.1182/blood-2014-04-570929 25145343 PMC4192751

[B45] Pan J. Li D. Xu Y. Zhang J. Wang Y. Chen M. (2017). Inhibition of Bcl-2/xl with ABT-263 selectively kills senescent type II pneumocytes and reverses persistent pulmonary fibrosis induced by ionizing radiation in mice. Int. J. Radiat. Oncol. Biol. Phys. 99, 353–361. 10.1016/j.ijrobp.2017.02.216 28479002 PMC6853175

[B46] Park C.-M. Oie T. Petros A. M. Zhang H. Nimmer P. M. Henry R. F. (2006). Design, synthesis, and computational studies of inhibitors of Bcl-XL. J. Am. Chem. Soc. 128, 16206–16212. 10.1021/ja0650347 17165773

[B47] Paulus A. Chitta K. Akhtar S. Personett D. Miller K. C. Thompson K. J. (2014). AT-101 downregulates BCL2 and MCL1 and potentiates the cytotoxic effects of lenalidomide and dexamethasone in preclinical models of multiple myeloma and Waldenström macroglobulinaemia. Br. J. Haematol. 164, 352–365. 10.1111/bjh.12633 24236538 PMC4406280

[B48] Peifer M. Fernández-Cuesta L. Sos M. L. George J. Seidel D. Kasper L. H. (2012). Integrative genome analyses identify key somatic driver mutations of small-cell lung cancer. Nat. Genet. 44, 1104 10.1038/ng.2396 22941188 PMC4915822

[B49] Petros A. M. Olejniczak E. T. Fesik S. W. (2004). Structural biology of the Bcl-2 family of proteins. Biochim. Biophys. Acta 1644, 83–94. 10.1016/j.bbamcr.2003.08.012 14996493

[B50] Reed J. C. Zha H. Aime-Sempe C. Takayama S. Wang H. G. (1996). “Structure-function analysis of Bcl-2 family proteins,” in Mechanisms of lymphocyte activation and immune regulation VI La Jolla:(Springer), 99–112. 8910675

[B51] Roberts A. W. Advani R. H. Kahl B. S. Persky D. Sweetenham J. W. Carney D. A. (2015). Phase 1 study of the safety, pharmacokinetics, and antitumour activity of the BCL2 inhibitor navitoclax in combination with rituximab in patients with relapsed or refractory CD20+lymphoid malignancies. Br. J. Haematol. 170, 669–678. 10.1111/bjh.13487 25942994 PMC4534314

[B52] Roberts A. W. Seymour J. F. Brown J. R. Wierda W. G. Kipps T. J. Khaw S. L. (2012). Substantial susceptibility of chronic lymphocytic leukemia to BCL2 inhibition: results of a phase I study of navitoclax in patients with relapsed or refractory disease. J. Clin. Oncol. 30, 488–496. 10.1200/jco.2011.34.7898 22184378 PMC4979082

[B53] Rudin C. M. Hann C. L. Garon E. B. Ribeiro de Oliveira M. Bonomi P. D. Camidge D. R. (2012). Phase II study of single-agent navitoclax (ABT-263) and biomarker correlates in patients with relapsed small cell lung cancer. Clin. Cancer Res. 18, 3163–3169. 10.1158/1078-0432.ccr-11-3090 22496272 PMC3715059

[B54] Schafer M. J. White T. A. Iijima K. Haak A. J. Ligresti G. Atkinson E. J. (2017). Cellular senescence mediates fibrotic pulmonary disease. Nat. Commun. 8, 14532 10.1038/ncomms14532 28230051 PMC5331226

[B55] Shamas-Din A. Kale J. Leber B. Andrews D. W. (2013). Mechanisms of action of Bcl-2 family proteins. Cold Spring Harb. Perspect. Biol. 5, a008714 10.1101/cshperspect.a008714 23545417 PMC3683897

[B56] Shangary S. Oliver C. L. Tillman T. S. Cascio M. Johnson D. E. (2004). Sequence and helicity requirements for the proapoptotic activity of Bax BH3 peptides. Mol. Cancer Ther. 3, 1343–1354. 15542773

[B57] Shi J. Zhou Y. Huang H.-C. Mitchison T. J. (2011). Navitoclax (ABT-263) accelerates apoptosis during drug-induced mitotic arrest by antagonizing Bcl-xL. Cancer Res. 71, 4518–4526. 10.1158/0008-5472.CAN-10-4336 21546570 PMC3129452

[B58] Shoemaker A. R. Mitten M. J. Adickes J. Ackler S. Refici M. Ferguson D. (2008). Activity of the Bcl-2 family inhibitor ABT-263 in a panel of small cell lung cancer xenograft models. Clin. Cancer Res. 14, 3268–3277. 10.1158/1078-0432.CCR-07-4622 18519752

[B59] Soderquist R. Pletnev A. A. Danilov A. V. Eastman A. (2014). The putative BH3 mimetic S1 sensitizes leukemia to ABT-737 by increasing reactive oxygen species, inducing endoplasmic reticulum stress, and upregulating the BH3-only protein NOXA. Apoptosis 19, 201–209. 10.1007/s10495-013-0910-y 24072590 PMC3947354

[B60] Soderquist R. S. Danilov A. V. Eastman A. (2014). Gossypol increases expression of the pro-apoptotic BH3-only protein NOXA through a novel mechanism involving phospholipase A2, cytoplasmic calcium, and endoplasmic reticulum stress. J. Biol. Chem. 289, 16190–16199. 10.1074/jbc.m114.562900 24778183 PMC4047389

[B61] Stamelos V. A. Robinson E. Redman C. W. Richardson A. (2013). Navitoclax augments the activity of carboplatin and paclitaxel combinations in ovarian cancer cells. Gynecol. Oncol. 128, 377–382. 10.1016/j.ygyno.2012.11.019 23168176

[B62] Tan J.-K. Then S.-M. Mazlan M. Raja Abdul Rahman R. N. Z. Jamal R. Wan Ngah W. Z. (2016). Gamma-tocotrienol acts as a BH3 mimetic to induce apoptosis in neuroblastoma SH-SY5Y cells. J. Nutr. Biochem. 31, 28–37. 10.1016/j.jnutbio.2015.12.019 27133421

[B63] Tahir S. K. Wass J. Joseph M. K. Devanarayan V. Hessler P. Zhang H. (2010). Identification of expression signatures predictive of sensitivity to the Bcl-2 family member inhibitor ABT-263 in small cell lung carcinoma and leukemia/lymphoma cell lines. Mol. Cancer Ther. 9, 545–557. 10.1158/1535-7163.mct-09-0651 20179162

[B64] Thanasopoulou A. Tzankov A. Schwaller J. (2014). Potent co-operation between the NUP98-NSD1 fusion and the FLT3-ITD mutation in acute myeloid leukemia induction. Haematologica 99, 1465–1471. 10.3324/haematol.2013.100917 24951466 PMC4562535

[B65] Tolcher A. W. Lorusso P. Arzt J. Busman T. A. Lian G. Rudersdorf N. S. (2015). Safety, efficacy, and pharmacokinetics of navitoclax (ABT-263) in combination with erlotinib in patients with advanced solid tumors. Cancer Chemother. Pharmacol. 76, 1025–1032. 10.1007/s00280-015-2883-8 26420235

[B66] Tse C. Shoemaker A. R. Adickes J. Anderson M. G. Chen J. Jin S. (2008). ABT-263: a potent and orally bioavailable Bcl-2 family inhibitor. Cancer Res. 68, 3421–3428. 10.1158/0008-5472.CAN-07-5836 18451170

[B67] Vaillant F. Merino D. Lee L. Breslin K. Pal B. Ritchie M. E. (2013). BCL-2 with the BH3 mimetic ABT-199 in estrogen receptor-positive breast cancer. Cancer Cell 24, 120–129. 10.1016/j.ccr.2013.06.002 23845444

[B68] Visvader M. Dinsdale D. Dyer M. J. S. Cohen G. M. (2009). Bcl-2 inhibitors: small molecules with a big impact on cancer therapy. Cell Death Differ. 16, 360–367. 10.1038/cdd.2008.137 18806758

[B69] Walaszczyk A. Dookun E. Redgrave R. Tual‐Chalot S. Victorelli S. Spyridopoulos I. (2019). Pharmacological clearance of senescent cells improves survival and recovery in aged mice following acute myocardial infarction. Aging Cell 18, e12945 10.1111/acel.12945 30920115 PMC6516151

[B70] Wan Ali W. A. S. R. Said M. Shahrir M. Shaharir S. S. Lin B. Yu A. (2015). A cross sectional study of cardiopulmonary complications and severity of pulmonary hypertension and lung fibrosis in patients with systemic sclerosis. Arch. Rheumatol. 30 (4), 311–318. 10.5606/archrheumatol.2015.5593

[B71] Willis S. N. Chen L. Dewson G. Wei A. Naik E. Fletcher J. I. (2005). Proapoptotic Bak is sequestered by Mcl-1 and Bcl-xL, but not Bcl-2, until displaced by BH3-only proteins. Genes Dev. 19, 1294–1305. 10.1101/gad.1304105 15901672 PMC1142553

[B72] Wilson W. H. O'connor O. A. Czuczman M. S. Lacasce A. S. Gerecitano J. F. Leonard J. P. (2010). Navitoclax, a targeted high-affinity inhibitor of BCL-2, in lymphoid malignancies: a phase 1 dose-escalation study of safety, pharmacokinetics, pharmacodynamics, and antitumour activity. Lancet Oncol. 11, 1149–1159. 10.1016/s1470-2045(10)70261-8 21094089 PMC3025495

[B73] Wong M. Tan N. Zha J. Peale F. V. Yue P. Fairbrother W. J. (2012). Navitoclax (ABT-263) reduces bcl-xL-mediated chemoresistance in ovarian cancer models. Mol. Cancer Therapeut. 11, 1026–1035. 10.1158/1535-7163.mct-11-0693 22302098

[B74] Yang I.-H. Jung J.-Y. Kim S.-H. Yoo E.-S. Cho N.-P. Lee H. (2019). ABT-263 exhibits apoptosis-inducing potential in oral cancer cells by targeting C/EBP-homologous protein. Cell. Oncol. 42, 357–368. 10.1007/s13402-019-00431-5 PMC12994282 30919222

[B75] Yang Y. Liu L. Naik I. Braunstein Z. Zhong J. Ren B. (2017). Transcription factor C/EBP homologous protein in health and diseases. Front. Immunol. 8, 1612 10.3389/fimmu.2017.01612 29230213 PMC5712004

[B76] Zhang H. Nimmer P. M. Tahir S. K. Chen J. Fryer R. M. Hahn K. R. (2007). Bcl-2 family proteins are essential for platelet survival. Cell Death Differ. 14, 943–951. 10.1038/sj.cdd.4402081 17205078

[B77] Zhu G. Deng Y. Pan L. Ouyang W. Feng H. Wu J. (2019). Clinical significance of the BRAFV600E mutation in PTC and its effect on radioiodine therapy. Endocr. Connect. 8, 754–763. 10.1530/ec-19-0045 31071680 PMC6547306

[B78] Zhu Y. Tchkonia T. Fuhrmann‐Stroissnigg H. Dai H. M. Ling Y. Y. Stout M. B. (2016). Identification of a novel senolytic agent, navitoclax, targeting the Bcl‐2 family of anti‐apoptotic factors. Aging Cell 15, 428–435. 10.1111/acel.12445 26711051 PMC4854923

